# Measles — United States, January 1–May 23, 2014

**Published:** 2014-06-06

**Authors:** Paul A. Gastañaduy, Susan B. Redd, Amy Parker Fiebelkorn, Jennifer S. Rota, Paul A. Rota, William J. Bellini, Jane F. Seward, Gregory S. Wallace

**Affiliations:** 1 Division of Viral Disease, National Center for Immunization and Respiratory Diseases, CDC

Measles is a highly contagious, acute viral illness that can lead to serious complications and death. Although measles elimination (i.e., interruption of year-round endemic transmission) was declared in the United States in 2000 (1), importations of measles cases from endemic areas of the world continue to occur, leading to secondary measles cases and outbreaks in the United States, primarily among unvaccinated persons (2). To update national measles data in the United States, CDC evaluated cases reported by states from January 1 through May 23, 2014. A total of 288 confirmed measles cases have been reported to CDC, surpassing the highest reported yearly total of measles cases since elimination (220 cases reported in 2011) (3). Fifteen outbreaks accounted for 79% of cases reported, including the largest outbreak reported in the United States since elimination (138 cases and ongoing). The large number of cases this year emphasizes the need for health-care providers to have a heightened awareness of the potential for measles in their communities and the importance of vaccination to prevent measles.

Confirmed measles cases in the United States are reported by state and local health departments to CDC using a standard case definition.* A measles case is considered confirmed if it is laboratory-confirmed or meets the clinical case definition (an illness characterized by a generalized rash lasting ≥3 days, a temperature of ≥101°F [≥38.3°C], and cough, coryza, and/or conjunctivitis) and is linked epidemiologically to a confirmed case. Measles cases are laboratory confirmed if there is detection in serum of measles-specific immunoglobulin M, isolation of measles virus, or detection of measles virus nucleic acid from a clinical specimen. Cases are considered imported if at least some of the exposure period (7–21 days before rash onset) occurred outside the United States and rash occurred within 21 days of entry into the United States, with no known exposure to measles in the United States during that time. An outbreak of measles is defined as a chain of transmission of three or more confirmed cases.

Patients with reported measles cases this year have ranged in age from 2 weeks to 65 years; 18 (6%) were aged <12 months, 48 (17%) were aged 1–4 years, 71 (25%) were aged 5–19 years, and 151 (52%) were aged ≥20 years. Forty-three (15%) were hospitalized, and complications have included pneumonia (five patients), hepatitis (one), pancytopenia (one), and thrombocytopenia (one). No cases of encephalitis and no deaths have been reported.

Measles cases have been reported from 18 states and New York City. Most cases were reported from Ohio (138), California (60), and New York City (26). Fifteen outbreaks have accounted for 227 (79%) of the 288 cases. The median outbreak size has been five cases (range: 3–138 cases). There is an ongoing outbreak involving 138 cases, occurring primarily among unvaccinated Amish communities in Ohio.

Of the 288 cases, 280 (97%) were associated with importations from at least 18 countries. The source of measles acquisition could not be identified for eight (3%) cases. Forty-five direct importations (40 U.S. residents returning from abroad and five foreign visitors) have been reported. Almost half (22 [49%]) of these importations were travelers returning from the Philippines, where a large outbreak has been occurring since October 2013. Imported cases were also associated with travel from other countries in the World Health Organization (WHO) Western Pacific Region (seven cases), as well as countries in the WHO South-East Asia (eight), European (four), Americas (three), and Eastern Mediterranean (one) regions. Measles genotype information was obtained from 103 (36%) of the 288 measles cases. Four measles virus genotypes were identified: B3 (67 cases), D9 (23), D8 (12), and H1 (one) (Table).

Most of the 288 measles cases reported this year have been in persons who were unvaccinated (200 [69%]) or who had an unknown vaccination status (58 [20%]); 30 (10%) were in persons who were vaccinated. Among the 195 U.S. residents who had measles and were unvaccinated, 165 (85%) declined vaccination because of religious, philosophical, or personal objections, 11 (6%) were missed opportunities for vaccination, and10 (5%) were too young to receive vaccination (Figure).

## Discussion

Measles elimination has been maintained in the United States since elimination was declared almost 15 years ago. However, approximately 20 million cases of measles occur each year globally, and importations into the United States continue to pose a risk for measles cases and outbreaks among unvaccinated persons. The 288 measles cases reported during January 1–May 23, 2014, including an ongoing outbreak involving 138 persons in Ohio, represent the highest number of measles cases reported for that period since 1994. The increase in measles this year serves as a reminder for health-care providers to be cognizant of the possibility of measles cases occurring in their communities.

Health-care providers should maintain a high suspicion for measles among febrile patients with rash. Patients with clinical symptoms compatible with measles (febrile rash plus cough, coryza, and/or conjunctivitis), should be asked about recent travel abroad and contact with returning travelers, and their vaccination status should be verified. Measles cases have been initially misdiagnosed as Kawasaki disease, dengue, and scarlet fever, among other diseases, underscoring the importance of considering measles in the differential diagnosis of clinically compatible cases. It is important to obtain viral specimens for confirmation and genotyping on any patient when measles is suspected, in addition to serology. Genetic characterization of measles virus can suggest the likely source of an imported virus. Because patients with measles often seek medical care, early recognition of suspected measles cases and implementation of appropriate infection control measures are vital to reduce transmission in health-care settings. Where possible, because of the high transmissibility of measles, patients with suspected measles should be promptly screened before entering waiting rooms and appropriately isolated (i.e., in an airborne isolation room or, if not available, in a separate room with the door closed), or have their office appointments scheduled at the end of the day to prevent exposure of other patients (4). To assist state and local public health departments with rapid investigation and control efforts to limit the spread of disease, suspected measles cases should be reported to local health departments immediately. State health departments should notify CDC about cases of measles within 24 hours of detection (5).

To date in 2014, a total of 40 importations have been reported among unvaccinated returning U.S. travelers. Among these, 22 acquired measles in the Philippines, where 32,030 measles cases (26,014 suspected cases and 6,016 confirmed cases) and 41 measles deaths have been reported from January 1 through April 20†. The large number of importations from the Philippines highlights how importations are related to increases in measles incidence in countries that are common destinations for U.S. travelers. Because measles remains endemic in countries in five out of the six WHO regions of the world, including India, from where six importations have occurred this year, the source of imported cases could be any country where measles continues to circulate. This underscores the importance of ensuring age-appropriate vaccination for all persons before international travel to any region of the world.

Health-care providers should remind persons who plan to travel internationally, including travel to large international events and gatherings (e.g., the 2014 FIFA World Cup in Brazil), of the increased risk for measles, § and encourage timely vaccination of all persons aged ≥6 months without evidence of measles immunity.¶ One dose of measles-mumps-rubella (MMR) vaccine is recommended for infants aged 6–11 months before travel, and 2 doses for persons aged ≥12 months, with a minimum interval between doses of 28 days (6).

In the three largest outbreaks of 2014, which account for over a half of all cases this year, transmission occurred after introduction of measles into communities with pockets of persons who were unvaccinated because of philosophical or religious beliefs. Although high population immunity throughout the United States (through maintaining ≥90% MMR vaccine coverage among children aged 19–35 months and adolescents) prevents spread from most importations (7,8), coverage varies at the local level, and unvaccinated children tend to cluster geographically, increasing the risk for outbreaks (9). Thus, maintaining high measles vaccination coverage is critical to prevent large measles outbreaks in the United States, and to protect and limit spread to infants too young to be vaccinated and to persons who cannot be vaccinated because of medical contraindications.

In the United States, routine MMR vaccination is recommended for all children, with the first dose given at age 12–15 months, and a second dose at age 4–6 years. Catch-up vaccination is recommended for children and adolescents who have not received 2 appropriately spaced doses. Unless they have other evidence of immunity, adults should receive at least 1 dose of MMR vaccine, and 2 appropriately spaced doses of MMR vaccine are recommended for health-care personnel, college students, and international travelers (6).

The findings in this report are subject to at least two limitations. First, underreporting might have occurred. Second, for a few cases complete data could not be ascertained (e.g., the source of infection). However, national surveillance is considered adequate to detect measles circulation in the United States in the postelimination era (10). These numbers are considered preliminary and are subject to change should additional details become available.

What is already known on this topic?

Measles elimination (i.e., interruption of year-round endemic transmission) has been maintained in the United States since 2000. Despite progress in global measles control, measles remains common in many countries of the world, and measles is imported regularly into the United States.

What is added by this report?

Both the highest number of measles cases and the largest outbreak since elimination have been reported to CDC this year. As of May 23, 2014, a total of 288 cases were reported, of which 258 (90%) were in persons who were unvaccinated or had unknown vaccination status. Forty (89%) of the 45 importations were associated with U.S. travelers returning from abroad.

What are the implications for public health practice?

Importations of measles into communities with unvaccinated persons can lead to measles cases and outbreaks in the United States. Maintenance of high vaccination coverage, ensuring timely vaccination before travel, and early detection and isolation of cases, are key factors to limit importations and the spread of disease.

Despite maintenance of measles elimination in the United States, importations from endemic countries continue to occur and have caused an unusually high number of measles cases in 2014. The most frequent sources of importations were unvaccinated U.S. travelers returning from abroad, with subsequent transmission among clusters of unvaccinated persons. Encouraging timely delivery of measles vaccination for persons traveling internationally and sustaining high vaccination coverage in the United States in accordance with the Advisory Committee on Immunization Practices (ACIP) routine immunization schedule are essential to limit measles importations and the spread of disease. To help expedite public health containment strategies, health-care providers should maintain a high awareness of measles, implement appropriate infection control measures when measles is suspected, and promptly report suspected cases to their local health departments.

## Figures and Tables

**FIGURE:**
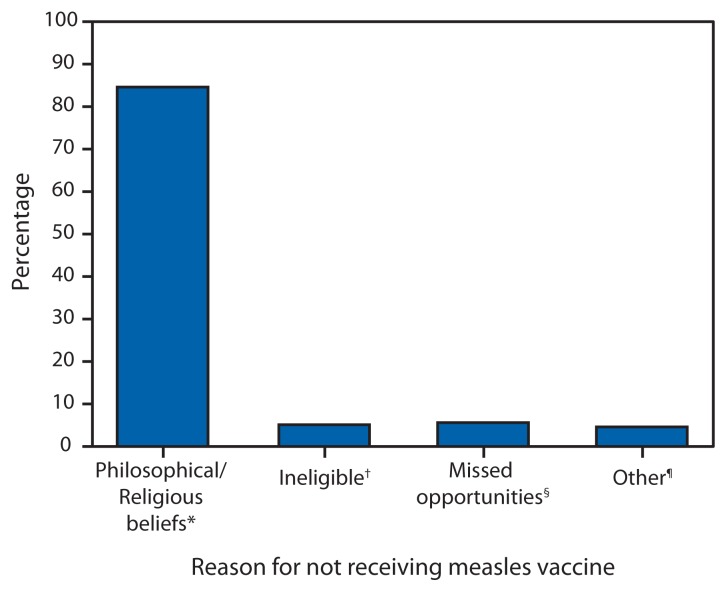
Percentage of U.S. residents with measles who were unvaccinated (N = 195), by reason for not receiving measles vaccine — United States, January 1–May 23, 2014 * Includes persons who were unvaccinated because of their own or their parents’ beliefs. ^†^ Includes person ineligible for measles vaccination, generally those aged <12 months. ^§^ Includes children aged 16 months–4 years who had not been vaccinated and international travelers aged ≥6 months who were unvaccinated but had no exemption. ^¶^ Includes persons who were known to be unvaccinated and the reason was unknown, and those who were born before 1957 and presumed to be immune.

**TABLE:** Countries associated with imported measles cases, by World Health Organization (WHO) region, number of cases (N = 45), and genotype — United States, January 1–May 23, 2014

WHO region	No. of cases	Country	No. of cases	Genotype*
African	0	—	—	
Eastern Mediterranean	1	Pakistan	1	B3
European	4	Dubai/Germany/England	1†	B3
		France/Belgium	1†	D8
		Netherlands	1	
		Republic of Georgia	1	B3
Americas	3	Brazil	1	B3
		Chile	1	D8
		Canada	1	D8
South-East Asia	8	India	6	D8
		Indonesia	1	
		Thailand/South Korea	1†	
Western Pacific	29	China	2	H1
		Micronesia	1	B3
		Philippines	22	B3, D9
		Saipan	1	B3
		Singapore	1	D8
		South-East Asia/Philippines	1†	
		Vietnam	1	D8

* Genotype was determined based on methodology described in the WHO measles virus nomenclature 2012 update: Wkly Epidemiol Rec 2012;87:73–80. Genotypes listed are those identified in a sample from the imported case or from a case that is epidemiologically linked to that importation.

† Patient had visited more than one country where measles is endemic during the incubation period, and exposure could have occurred in any of the countries and regions listed.

## Footnotes

* Available at http://www.cdc.gov/vaccines/pubs/surv-manual/chpt07-measles.pdf.

† Additional information available at http://www.wpro.who.int/immunization/documents/MRBulletinVol8Issue04.pdf?ua=1.

§ Additional information available at http://wwwnc.cdc.gov/travel/notices.

¶ Presumptive evidence of measles immunity: 1) documentation of age-appropriate vaccination with a live measles virus–containing vaccine (preschool-aged children: 1 dose; school-aged children [grades K–12]: 2 doses; adults not at high risk: 1 dose); or 2) laboratory evidence of immunity; or 3) laboratory confirmation of disease; or 4) born before 1957.

## References

[1] Katz SL, Hinman AR (2004). Summary and conclusions: Measles elimination meeting, 16–17 March 2000. J Infect Dis.

[2] Fiebelkorn AP, Redd SB, Gallagher K (2010). Measles in the United States during the Postelimination Era. J Infect Dis.

[3] CDC (2013). Summary of notifiable diseases—United States, 2011. MMWR.

[4] CDC (2007). 2007 guideline for isolation precautions: preventing transmission of infectious agents in healthcare settings.

[5] Council of State and Territorial Epidemiologists (2012). List of nationally notifiable conditions.

[6] CDC (2013). Prevention of measles, rubella, congenital rubella syndrome, and mumps: 2013 summary recommendations of the Advisory Committee on Immunization Practices (ACIP). MMWR.

[7] CDC (2013). National, state, and local area vaccination coverage among children aged 19–35 months—United States, 2012. MMWR.

[8] CDC (2012). Documentation and verification of measles, rubella, and congenital rubella syndrome elimination in the Region of the Americas.

[9] Smith PJ, Chu SY, Barker LE (2004). Children who have received no vaccines: who are they and where do they live?. Pediatrics.

[10] Harpaz R, Papania MJ, McCauley MM, Redd SB (2004). Has surveillance been adequate to detect endemic measles in the United States?. J Infect Dis.

